# Frequency and Characteristics of Trials Using Medical Writer Support in High-Impact Oncology Journals

**DOI:** 10.1001/jamanetworkopen.2022.54405

**Published:** 2023-02-01

**Authors:** Eva Buck, Alyson Haslam, Jordan Tuia, Vinay Prasad

**Affiliations:** 1 Department of Epidemiology and Biostatistics, University of California, San Francisco

## Abstract

**Importance:**

The practice of using medical writers to communicate scientific information has gained popularity, but it may affect how and what information is communicated.

**Objective:**

To assess characteristics of oncology trials that use medical writers and whether there is an association between the use of medical writers and trial success or the primary outcome evaluated.

**Design, Setting, and Participants:**

This cross-sectional study included oncology trials testing a tumor-targeting intervention that were published in *The Lancet*, *The Lancet Oncology*, *JAMA*, *JAMA Oncology*, *Journal of Clinical Oncology*, and *The New England Journal of Medicine* between May 1, 2021, and May 1, 2022.

**Exposures:**

Assistance of medical writers or no assistance.

**Main Outcomes and Measures:**

The main outcomes were the percentage of studies with medical writers, the percentage of trial successes reported with medical writers, the association between trial success and medical writer use, and the association between a primary end point and medical writer use.

**Results:**

Among 270 studies, 141 (52.2%) included a medical writer and 129 (47.8%) did not include a medical writer. Of the studies that included a medical writer, 83 (58.9%) were successful. Of the studies that did not include a medical writer, 64 (49.6%) were successful (*P* = .16 for difference). Studies with medical writers were less likely than studies without medical writers to have the end point of overall survival (15 [10.6%] vs 17 [13.2%]) and disease-free or event-free survival (16 [11.3%] vs 29 [22.5%]), whereas studies with a medical writer were more likely to have the end point of progression-free survival (32 [22.7%] vs 17 [13.2%]). Use of medical writer was associated with the conclusions being presented favorably in all studies (113 [80.1%] vs 89 [69.0%]; odds ratio [OR], 1.81 [95% CI, 1.04-3.19]), but when adjusted for other variables, there was no association (OR, 1.84 [95% CI, 0.92-3.72]).

**Conclusions and Relevance:**

In this cross-sectional study, trials using medical writers were more likely to report surrogate end points, such as progression-free survival, and favorable conclusions, but when adjusted for trial phase, randomization, and study funding, there was no association with favorable conclusions. These findings suggest that journals need heightened scrutiny for studies with medical writers and that authorship should be properly acknowledged.

## Key Points

**Question:**

What are the characteristics of cancer studies that use and do not use medical writer support?

**Findings:**

In this cross-sectional analysis of 270 clinical trials, compared with studies that did not use medical writers, studies with medical writers were more likely to focus on progression-free survival than overall survival and were more likely to report favorable conclusions, but there was no association with favorable conclusions in the adjusted analysis.

**Meaning:**

These findings suggest that the use of medical writers is associated with the end point of progression-free survival.

## Teaser

This cross-sectional study reviews original oncology trials to assess whether the declared use of medical writers is associated with trial success and the use of a particular type of end point.

## Introduction

The global medical writing market size was $3.6 billion in 2021 and is projected to rise to the value of $8.4 billion by 2030.^1^ Medical writers may be employed by pharmaceutical companies or third-party agencies and work alongside physicians, scientists, and medical professionals to draft and edit articles for publication and to assist with information dissemination and documentation.

Companies and researchers state they rely on medical writers to save time and improve the quality of a manuscript, thereby having a higher chance of having published articles compared with those who do not use a medical writer.^2^ However, certain ethical issues arise when using medical writers.

Third-party medical writers may lack accountability for the results and conclusions of publications because they do not qualify for authorship, and when they are listed as authors, they are unlikely to report potential conflicts of interest that might bias study results.^3^ Further, because their livelihood depends on getting funding from companies in industry, they may be financially incentivized to present findings in a way that is favorable to the company paying them, as has been shown in other areas of research.^4^

Issues around authorship may arise. The International Committee of Medical Journal Editors defines authorship as substantial contributions to the design, data acquisition, analysis, interpretation, and drafting of the manuscript, giving approval for the final version, and accountability for all aspects of the work.^5^ Overreliance on medical writers might threaten these standards.

The term *medical writer* may be considered a rebranding of the term *ghostwriter*, the former term suggesting a significantly lower contribution to the manuscript.^6^ Despite acknowledgment of medical writing and editorial assistance in footnotes, the use of medical writers allows for medical manuscripts to be written without sufficient disclosure of how the manuscript was composed. In the present study, we reviewed original oncology trials to assess whether the declared use of medical writers was associated with trial success and the use of a particular type of end point.

## Methods

In accordance with 45 CFR §46.102(f), this cross-sectional study was not submitted for institutional review board approval because it involved publicly available data and did not involve individual patient data. This study was not preregistered since our objective was hypothesis generating, rather than confirmatory. Our study adhered to Strengthening the Reporting of Observational Studies in Epidemiology (STROBE) guideline.

### Article Search

We searched 6 top medical and oncology journals (*The Lancet*, *The Lancet Oncology*, *JAMA*, *JAMA Oncology*, *The New England Journal of Medicine*, and *Journal of Clinical Oncology*) for original cancer trials. We selected these journals because they are high-impact journals for both general medicine and the medical subdiscipline of oncology that publish human randomized clinical trials. Two of us (A.H. and V.P.) have used these journals in a previous study.^7^

We searched for studies published between May 1, 2021, and May 1, 2022. Included studies needed to (1) report on a cancer trial, (2) report original research, and (3) evaluate a tumor-targeting treatment. Excluded studies (1) had a primary end point of noninferiority or equivalence, (2) were a meta-analysis or retrospective analysis, (3) were an observational study, (4) were not cancer trials, and/or (5) had a non–tumor targeting intervention. We allowed multiple articles on the same trial, as long as they published or presented different aspects of the trial (eg, different titles, different outcomes). Articles were collected manually from journal websites.

### Data Abstraction and Defining Variables

We abstracted data on the journal, trial phase, date, study design, masking, tumor type, and primary end point(s) and/or outcome(s) of both the overall trial and the published manuscript; disclosure of medical writers, name(s) of medical writer(s), number of medical writers, medical writing company name(s), and number of medical writing companies; disclosure of English language editing; whether the intervention included radiotherapy, surgery, or drug(s); name of drug(s) used in the intervention; a statement of adherence to a research checklist (eg, Consolidated Standards of Reporting Trials or Transparent Reporting of Evaluations with Nonrandomized Designs); study funding; and the drug’s manufacturer(s). Medical writers were identified in the Acknowledgments or Funding section of a journal article with the key words *medical writing support*, *editorial support*, *writing support*, *medical editing*, *medical writing assistance*, *editorial assistance*, *assisted with preparing the final manuscript*, *assistance in preparation of the article*, and *provided drafts*.

We coded study success by whether the study met its primary end point of the study publication (met vs nonmet), as reported in the trial publication. This was sometimes different than the primary end point of the trial (eg, quality of life in publication vs overall survival [OS] in the trial) We also coded the tone of the authors’ conclusion (positive vs negative and/or equivocal). For single-arm trials, we determined study success based on the value used for the sample size calculation (eg, the alternate hypothesis). Studies with no clearly defined end point benchmark or studies with multiple outcomes with differential effects were coded as having an equivocal study end point. The coding was done by 2 of 3 reviewers, and disagreements were adjudicated by a third (E.B., A.H., and/or J.T.).

### Statistical Analysis

We calculated the number of studies with or without medical writers for each of the following: tumor type (breast, colorectal, non–small cell lung cancer, or other), trial phase (1, 2, 3, 4, or not indicated), randomization (randomized or nonrandomized), blinding status (double-blind, single-blind, open-label, or not indicated), primary end point (progression-free survival [PFS], OS, disease-free survival [DFS], and other), whether the study end point was met (yes vs no or equivocal), intervention type (drug, radiotherapy, or surgery), and journal. We used χ^2^ and Wilcoxon rank sum tests to assess statistical differences in categorical and continuous variables between studies that used a medical writer and studies that did not use a medical writer. Specifically, our primary aim was to test whether studies that used medical writers were more likely to be reported as successful than studies that did not use medical writers. Our secondary aim was to test whether studies that used a medical writer were more likely to use a particular type of outcome (eg, PFS rather than OS) than studies that did not use medical writers. To control for an association between medical writers and study success or author conclusion, we performed 2 logistic regression models with success and conclusion as the outcomes. We included study characteristics in the model and removed them 1 at a time if their removal resulted in a lower Akaike information criterion. We forced in the variable indicating the use of a medical writer in both models. We used Excel, version 2022 (Microsoft Corporation) and R statistical software, version 4.2.1 (R Program for Statistical Computing) for all analysis and a 2-sided *P* value of less than 0.05 as our threshold for statistical significance.

## Results

**Figure 1.**
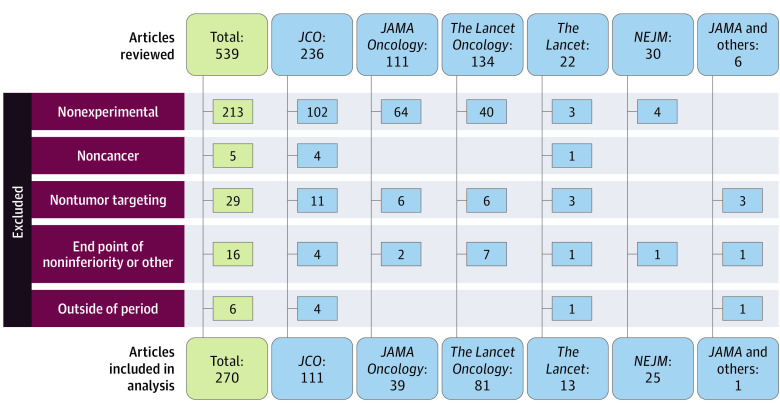
Flowchart of Oncology Articles Published in Top Medical and Oncology Journals Selected for Medical Writer Analysis *JCO* indicates *Journal of Clinical Oncology*; *NEJM*, *The New England Journal of Medicine*.

Our search identified 539 articles. After excluding articles not meeting our inclusion criteria, we identified 270 articles (Figure 1). For the 270 unique studies, there were 198 different medical writers and 40 different medical writing companies. Characteristics of these studies, stratified by whether medical writers assisted in the writing of the manuscript or not, are presented in Table 1.

**Table 1.** Characteristics of Oncology Interventional Studies in Top Medical and Oncology Journals Stratified by Whether the Studies Were Written With the Assistance of Medical Writers

Characteristic	Studies, No. (%) (N = 270)^a^		*P* value
	Did not use medical writers	Used medical writers	
All studies	129 (47.8)	141 (52.2)	NA
Journal			
* JAMA*	0	1 (0.7)	<.001
* JAMA Oncology*	28 (21.7)	11 (7.8)	
* Journal of Clinical Oncology*	71 (55.0)	40 (28.4)	
* The Lancet Oncology*	24 (18.6)	57 (40.4)	
* The Lancet*	2 (1.6)	11 (7.8)	
* The New England Journal of Medicine*	4 (3.0)	21 (14.9)	
Randomized	82 (63.6)	95 (67.4)	.60
Trial phase			
1	7 (5.4)	7 (5.0)	.10
2	61 (47.3)	51 (36.2)	
3	58 (45.0)	83 (58.9)	
4	1 (0.8)	0	
Not available	2 (1.6)	0	
Blinding status			
No			<.001
Open-label	115 (89.1)	104 (73.8)	
Single-blinded	4 (3.1)	1 (0.7)	
Double-blinded	10 (7.8)	36 (25.5)	
Tumor type			
Brain or CNS	7 (5.4)	2 (1.4)	.02
Breast	22 (17.1)	13 (9.2)	
Cervical	1 (0.8)	4 (2.8)	
CRC	7 (5.4)	6 (4.3)	
Endometrial	1 (0.8)	2 (1.4)	
Esophageal	0	1 (0.7)	
Gastrointestinal tract	4 (3.1)	8 (5.7)	
Hematologic	21 (16.3)	16 (11.3)	
Hepatobiliary	4 (3.1)	8 (5.7)	
HNSCC	8 (6.2)	3 (2.1)	
Lung	15 (11.6)	17 (12.1)	
Lymphoma	8 (6.2)	13 (9.2)	
Melanoma	3 (2.3)	7 (5.0)	
Myeloma	1 (0.8)	0	
Other	4 (3.1)	5 (3.5)	
Ovarian	4 (3.1)	8 (5.7)	
Pancreatic	5 (3.9)	0	
Prostate	5 (3.9)	11 (7.8)	
Sarcoma	4 (3.1)	1 (0.7)	
Thyroid	0	3 (2.1)	
Urothelial and/or RCC	5 (3.9)	12 (8.5)	
Primary end point			
DFS or EFS	29 (22.5)	16 (11.3)	.009
Response rate	23 (17.8)	32 (22.7)	
OS	17 (13.2)	15 (10.6)	
PFS	17 (13.2)	32 (22.7)	
Quality of life	6 (4.7)	7 (5.0)	
Safety and/or tolerability	11 (8.5)	9 (6.4)	
Multiple	13 (10.1)	26 (18.4)	
Other	13 (10.1)	4 (2.8)	
Primary end point met			
Yes	64 (49.6)	83 (58.9)	.16
No or equivocal	65 (50.4)	58 (41.1)	
Author conclusion			
Positive	89 (69.0)	113 (80.1)	.049
Negative or equivocal	40 (31.0)	28 (19.9)	
Funding			
Industry	70 (54.3)	131 (92.9)	<.001
Nonindustry	59 (45.7)	10 (7.1)	
Statement about adhering to a research checklist	21 (16.3)	8 (5.7)	.009

Abbreviations: CNS, central nervous system; CRC, colorectal cancer; DFS, disease-free survival; EFS, event-free survival; HNSCC, head and neck squamous cell carcinoma; OS, overall survival; PFS, progression-free survival; RCC, renal cell carcinoma.

^a^ Data were acquired between March 1, 2021, and March 1, 2022. Percentages have been rounded and might not total 100.

Of the 270 studies, 141 (52.2%) included a medical writer and 129 (47.8%) did not include a medical writer. The most common tumor types in studies with medical writers were hematologic (16 [11.3%]), lung (17 [12.1%]), breast (13 [9.2%]), and urothelial and/or renal (12 [8.5%]). The most common tumor types in studies without medical writers were breast (22 [17.1%]), hematologic (21 [16.3%]), and lung (15 [11.6%]).

**Figure 2.**
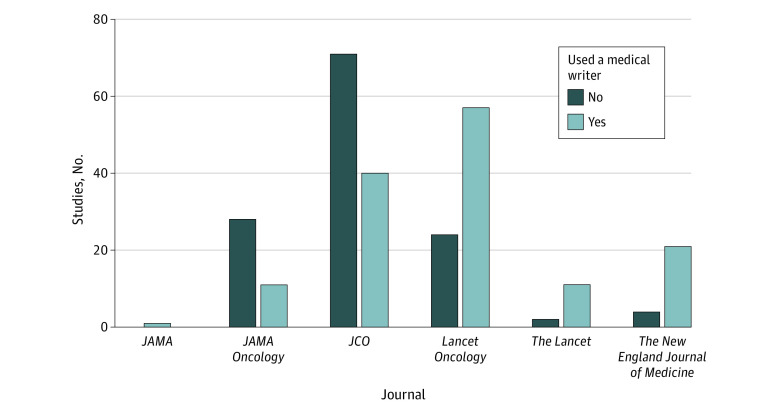
Number of Oncology Studies Published in High-Impact Medical and Oncology Journals by Whether the Study Used a Medical Writer *JCO* indicates *Journal of Clinical Oncology.*

There were differences in blinding status between studies that used a medical writer and studies that did not, with studies using a medical writer being more likely to be blinded than studies that did not use a medical writer (36 [25.5%] vs 10 [7.8%]; *P* < .001). Compared with studies without medical writers (Table 1 and Figure 2), the studies with medical writers were more likely to be published in *The Lancet Oncology* (57 [40.4%] vs 24 [18.6%]; *P* < .001). The studies with medical writers were less likely to be published in the *Journal of Clinical Oncology* (40 [28.4%] vs 71 [55.0%]). Studies with medical writers were less likely to include surgery (3 [2.1%] vs 17 [13.2%]; *P* = .001) or radiotherapy (9 [6.4%] vs 34 [26.4%]; *P* < .001) as part of the intervention compared with studies without medical writers.

**Figure 3.**
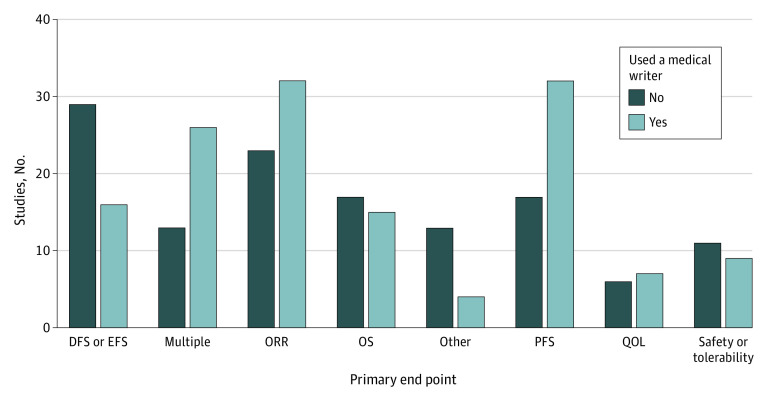
Number of Studies for Each End Point, by Whether the Study Used a Medical Writer, for Oncology Studies Reported in High-Impact Journals DFS indicates disease-free survival; EFS, event-free survival; ORR, overall response rate; OS, overall survival; PFS, progression-free survival; and QOL, quality of life.

The most common end point for a study using a medical writer was PFS, occurring in 32 studies (22.7%). The most common end point for a study without a medical writer was DFS or event-free survival, occurring in 29 studies (22.5%). Compared with studies that did not use medical writers, studies with medical writers were less likely to have the end point of OS (17 [13.2%] vs 15 [10.6%]) and DFS (29 [22.5%] vs 16 [11.3%]), whereas studies with a medical writer were more likely than studies without medical writers to have the end point of PFS (32 [22.7%] vs 17 [13.2%]; *P* = .001 for global differences) (Figure 3).

Of the studies that included a medical writer, 83 (58.9%) were successful (ie, met the primary end point of the study). Of the studies that did not include a medical writer, 64 (49.6%) were successful (odds ratio [OR], 1.45 [95% CI, 0.90-2.36]). Of the studies that included a medical writer, 113 (80.1%) had positive conclusions. Of the studies that did not include a medical writer, 89 (69.0%) had positive conclusions (OR, 1.81 [95% CI, 1.04-3.19]). When adjusted for study funding, randomization, trial phase, and being a primary report of the study, the use of a medical writer was not associated with meeting the study end point (OR, 1.04 [95% CI, 0.60-1.81). Similarly, when adjusted for other study factors, the use of a medical writer was not associated with positive author conclusions (OR, 1.84 [95% CI, 0.92-3.72]). Of the studies that used medical writers, 25 (17.7%) used medical writers who worked for the sponsoring company of the study, 100 (70.9%) included medical writers who worked for a medical writing company, and 8 (5.7%) used medical writers who worked for a hospital or other group.

One-hundred eighty-one of the 202 medical writers (89.6%) appeared in only 1 study, 18 (8.9%) appeared in 2 studies, and 3 (1.5%) appeared in 3 or 4 studies. Of the 82 studies using only 1 medical writer with only 1 appearance overall, 44 (53.6%) were successful. Of the 44 studies that used 2 medical writers, 28 (63.6%) were successful. Of the 13 studies that used 3 medical writers, 9 (69.2%) were successful. Of the 2 studies that used 4 medical writers, 2 (100%) were successful (*P* = .046 for global differences) (Table 2). The rates of success for each company are presented in the eTable in Supplement 1.

**Table 2.** Distribution of Medical Writers per Study and the Probability of Meeting the Primary End Point in Medical Oncology Studies Published in High-Impact Journals

Distribution	Total No. of studies using medical writers	Studies, No. (%)	
		Met primary end point	Favorable conclusions presented
Medical writers per study, No.			
4	2	2 (100)	2 (100)
3	13	9 (69.2)	11 (84.6)
2	44	28 (63.6)	34 (77.3)
1	82	44 (53.6)	66 (80.5)
Medical writing companies per study, No.			
2	7	6 (85.7)	7 (100)
1	120	74 (61.7)	96 (80.0)

## Discussion

Our analysis of 270 high-impact cancer articles and their use of medical writers revealed several key findings. First, oncology studies with medical writers were more likely to have favorable conclusions than studies without medical writers. However, there was no association when adjusted for study funding. Study and author funding can lead to conflicts of interest, which can also lead to bias in study conclusions.^4,8,9^ Second, we found that studies with medical writers were more likely to have the end point of PFS, while studies without medical writers were more likely to have the end point of OS. These findings suggest that medical writers are recruited for trials that focus on end points of lesser importance.

We found that studies with medical writers were more likely to investigate the end point of PFS and objective response rate, which are common surrogate end points. Surrogate end points often fail to estimate which therapies improve survival.^10^ An intervention that achieves improvement in a surrogate end point, with toxic effects and cost, and has yet to show improvement in quality of life or survival may be debated by physicians. Use of a medical writer may help assuage or distract from such concerns.

We found that studies that did not use medical writers were more likely to provide a statement of adherence to a reporting checklist. While it was beyond the scope of our study to assess the completeness of reporting according to established checklists, another study^11^ found that use of a medical writer was associated with more complete reporting of checklist components; however, that study did not report declared adherence.

A medical writer may draft a manuscript without a major contribution being shown in the official author list. The International Committee of Medical Journal Editors has clear authorship guidelines. To qualify for authorship, one must have made substantial contributions to the conception or design of the work. They must also draft the work or revise it critically for important intellectual content.^5,12^ Our analysis is limited as we did not have access to versions of documents with tracked changes and thus could not delineate appropriate authorship criteria.

Medical writers are often cited as assisting in the preparation of the manuscript or first draft.^12^ It may be the case that a medical writer has drafted a manuscript predominantly and is acknowledged in a footnote, while the official authors did not contribute as heavily. A concern is that when medical writers are not listed as authors, they are not held accountable for the information presented in the publication.^12^ If medical writers are influential in the drafting of a manuscript, including the use of language and even trial outcomes, perhaps they should be listed as authors or restricted from involvement in publications.

In a previous study,^11^ 47% of medical publications were confirmed as eligible for inclusion in a group of articles with medical writing support, although for ghostwriting, which can have varied definitions, the estimate has varied widely, from 1% to 91%.^13^ These estimates may vary depending on study design, year of publication, and biomedical discipline. Our finding of 52.2% is representative of how often medical writers were generally used in contemporary oncology research, but this percentage is projected to increase as the global medical writing market size continues to grow.^1^

### Strengths and Limitations

Our study has several strengths. Our study is the first, to our knowledge, to examine the prevalence of medical writing in oncology studies, and we are the first, to our knowledge, to examine the association of medical writing assistance with whether studies met the primary end point or had favorable conclusions.

This study also has limitations. The determination of favorable conclusions could be subjective and, to an extent, so could meeting the study’s end point since some studies did not indicate the primary end point or were nonrandomized studies without a comparison arm. To minimize these biases, we had these 2 variables double coded and we used the study outcome that was used for the sample size determination. Our results may not be generalizable to the oncology literature at large. We noted differences in outcomes between journals, and because we did not comprehensively examine studies in all journals, we cannot rule out that studies published in different journals would have different results. It may be that some medical writers were not identified in the publications, which may have biased our results toward the null hypothesis. In addition, we were unable to quantify the contribution that each medical writer made to the manuscript, only that they were used in the drafting of the manuscript.

## Conclusions

In this cross-sectional study, original oncology trials using medical writers were less likely than trials without medical writers to report OS but were more likely to report PFS as an outcome. They were also more likely to report favorable conclusions, but when adjusted for other factors, there was no association. These findings suggest that journals need to give more scrutiny to studies with medical writers and that authorship needs to be properly acknowledged.

## Notes

Supplement 1. **eTable.** Names of Medical Writing Companies and Success Rate of Oncology Studies Who Used These Companies

Supplement 2.
Data Sharing Statement
