# Supplementary material for: Frequency and Characteristics of Trials Using Medical Writer Support in High-Impact Oncology Journals
Source: JAMA Netw Open. 2023 Feb 1;6(2):e2254405. doi: 10.1001/jamanetworkopen.2022.54405 (PMC9892954; doi:10.1001/jamanetworkopen.2022.54405)
Supplement: Supplement 1. — eTable. Names of Medical Writing Companies and Success Rate of Oncology Studies Who Used These Companies [file jamanetwopen-e2254405-s001.pdf]

Supplementary Online Content

Buck E, Haslam A, Tuia J, Prasad V. Frequency and characteristics of trials using medical writer support in high-impact oncology journals. *JAMA Netw Open*. 2023;6(2):e2254405. doi:10.1001/jamanetworkopen.2022.54405

eTable. Names of Medical Writing Companies and Success Rate of Oncology Studies Who Used These Companies

This supplementary material has been provided by the authors to give readers additional information about their work.

©2023 Buck E et al. *JAMA Network Open.*

eTable. Names of Medical Writing Companies and Success Rate of Oncology Studies Who Used These Companies

Company Name	Success Rate, n (%)
Parexel	11/12 (91.67)
ApotheCom	8/9 (88.89)
Ashfield MedComms	7/7 (100.00)
Oxford PharmaGenesis	5/7 (71.43)
OPEN Health company	4/5 (80.00)
ICON	4/4 (100.00)
Fishawack Communications	3/4 (75.00)
Mudskipper Business	3/4 (75.00)
Eloquent Scientific Solutions	3/3 (100.00)
Nexus Global Group Science	3/3 (100.00)
Health Interactions	2/3 (66.67)
Healthcare Consultancy Group	1/3 (33.33)
Cello Health Communications/MedErgy	2/2 (100.00)
CMC AFFINITY, McCann Health Medical Communications	2/2 (100.00)
Complete HealthVizion, McCann Health Medical Communications	2/2 (100.00)

Company Name	Success Rate, n (%)
Elevate Medical Affairs	2/2 (100.00)
Excel Medical Affairs	2/2 (100.00)
Novartis	2/2 (100.00)
CStone Pharmaceuticals	2/2 (100.00)
Prime	2/2 (100.00)
Bio Connections LLC	1/2 (50.00)
Paragon	1/2 (50.00)
ClinicalThinking	0/2 (0)
Agenus	1/1 (100.00)
Amgen	1/1 (100.00)
Articulate Science	1/1 (100.00)
Asan Medical Center	1/1 (100.00)
BOLDSCIENCE Inc	1/1 (100.00)
Chrysalis Medical Communications	1/1 (100.00)
Clovis Oncology	1/1 (100.00)
Immunocore	1/1 (100.00)
Innovent Biologics	1/1 (100.00)
IQVIA	1/1 (100.00)
Janssen	1/1 (100.00)
JetPub Scientific Communications	1/1 (100.00)
Kite	1/1 (100.00)
Lukasz Wujak MedComms	1/1 (100.00)
MedErgy	1/1 (100.00)
Medical Communication Company	1/1 (100.00)
MediTech Media	1/1 (100.00)
MedVal Scientific Information Services, LLC	1/1 (100.00)

©2023 Buck E et al. *JAMA Network Open.*

Company Name	Success Rate, n (%)
Miller Medical Communications	1/1 (100.00)
MMS Holdings	1/1 (100.00)
Nucleus Global	1/1 (100.00)
Seagen	1/1 (100.00)
Unicancer	1/1 (100.00)
Cactus Life Sciences	0/1 (0)
Eli Lilly and Company	0/1 (0)
ABCSG	0/1 (0)
Medi-Kelsey Limited	0/1 (0)
Cactus Life Sciences	0/1 (0)
NSABP	0/1 (0)
ProScribe—Envision Pharma Group	0/1 (0)
Scion	0/1 (0)
Sotio	0/1 (0)
Twist Medical	0/1 (0)

©2023 Buck E et al. *JAMA Network Open.*
