# Supplementary material for: Frequency and Characteristics of Trials Using Medical Writer Support in High-Impact Oncology Journals
Source: JAMA Netw Open. 2023 Feb 1;6(2):e2254405. doi: 10.1001/jamanetworkopen.2022.54405 (PMC9892954; doi:10.1001/jamanetworkopen.2022.54405)
Supplement: Supplement 2. — Data Sharing Statement [file jamanetwopen-e2254405-s002.pdf]

Data Sharing Statement

Buck. Frequency and Characteristics of Trials Using Medical Writer Support in High-Impact Oncology Journals. *JAMA Netw Open*. Published February 01, 2023. doi:10.1001/jamanetworkopen.2022.54405

Data
Data available: No

Additional Information
Explanation for why data not available: Data are publicly available
